# Nephrologists and patients: between vulnerability and equity

**DOI:** 10.1590/2175-8239-JBN-2019-0060

**Published:** 2019-09-12

**Authors:** Fábio Humberto Ribeiro Paes Ferraz, Cibele Isaac Saad Rodrigues, Giuseppe Cesare Gatto, Natan Monsores de Sá

**Affiliations:** 1 Fundação de Ensino e Pesquisa em Ciências da Saúde, Escola Superior de Ciências da Saúde, Departamento de Graduação em Medicina, Brasília, DF, Brasil.; 2 Pontificia Universidade Católica de São Paulo. Faculdade de Ciências Médicas e da Saúde, Departamento de Medicina, Programa Mestrado Profissional em Educação nas Profissões de Saúde, Sorocaba, SP, Brasil.; 3 Universidade de Brasília, Hospital Universitário de Brasília, Unidade de Transplante, Brasília, DF, Brasil.; 4 Universidade de Brasília, Faculdade de Ciências da Saúde, Cátedra Unesco de Bioética, Brasília, DF, Brasil.

Dear editor

The word “vulnerability” derives from the Latin term *vulnerabilis*, or from the Greek *vulnus*, and means “to hurt”.^1^ It was first added to the field of Bioethics in 1978, after the publication of the document called Belmont Report, which aimed to outline the ethical principles guiding research with human beings in the United States.^1^

Since then, the term “vulnerable” has been used in caring ethics, and can be attributed to individuals, patients, family members, caregivers, health professionals or populations that are incapable of consent and/or reduced autonomy.

The vulnerability among chronic renal patients is multifactorial and multi-faceted, as these patients are deprived of their autonomy, requiring the availability, access and adherence to various forms of renal replacement therapy (RRT) for the maintenance of one’s life.

In this context, Obregón *et al.’s* 2 paper is quite original, since it reverses the traditional viewpoint, concentrating not on the vulnerability of the patient, but rather on nephrologists themselves, regarding the technological, economic and professional contingencies that affect their field of work, their medical fees and their labor ties.^2^

A recent paper produced by our group sought to expand the view on vulnerability in the developing countries that make up the BRICS (Brazil, Russia, India, China and South Africa).^3^These countries account for 40% of the world population, 25% of the World’s Gross Product and 40% of the Global Burden of Disease.^3^ Through documentary analysis and systematic bibliographic research, the objective was to analyze the main bioethical issues in accessing the various forms of RRT in these countries.

Regarding renal transplantation, there were permissive legislation on organ tourism (South Africa), the occurrence of renal transplants with deceased donors without prior consent (China), a high number of kidney transplants involved with evidence of commercialization of organs (India), difficulty concerning extra-official data in the international literature (Russia) and regional disparities in access to renal transplantation (in all).^3^ About dialysis, we find the most sensitive bioethical issues: prioritization of dialysis only for patients eligible for renal transplantation (South Africa), lack of government funding for dialysis and high-cost drugs (India), and inequalities in the provision of dialysis (all).^3^

While equity is a bioethical concept distinct from vulnerability, based on the Aristotelian premise that “unequal must be treated unequally,” promoting a fair provision of RRT to the most vulnerable countries, especially developing countries, would bring an improvement to the individuals’ vulnerabilities, as a bioethical reference, both for patients (due to better accessibility to treatment) and for nephrologists themselves (who often need to make difficult ethical choices in the context of scarce resources). The “Kidney Health for All” theme for the celebrations for the 2019’s World Kidney Day is an invitation to such a reflection. In fact, issues related to inequality in access to renal health are increasingly common in the world literature.^5^
